# Towards the sustainable development of logistics system model: A system dynamics approach

**DOI:** 10.1371/journal.pone.0279687

**Published:** 2023-01-26

**Authors:** Xuehua Ji, Yue Zhai, Shaochuan Fu, Changxiang Lu

**Affiliations:** 1 School of Economics and Management, Beijing Jiaotong University, Beijing, PR China; 2 Research Center for Central and Eastern Europe, Beijing Jiaotong University, Beijing, PR China; 3 Institute of Public Health & Emergency Management, Taizhou University, Taizhou, Zhejiang, China; 4 Business Colleage, Taizhou University, Taizhou, Zhejiang, China; Institute for Advanced Sustainability Studies, GERMANY

## Abstract

The contradiction between the limited service capacity of system and the explosive growth of demand has hampered the sustainable development of logistics system. Taking into account the structure of logistics system, this study introduces a system dynamics approach to explore the complex correlation and coupling structure of system, analyzes the multiple feedback loops and design the different scenarios. Results show that the validity and rationality of logistics system model, and the error percentage of GDP and logistics demand factors less than 6%. The influence of the investment in reverse logistics, logistics management, information and organizational management factor on the service quality of logistics system increases in turn. Additionally, adjustment of industrial structure has a significant impact on the investment in information management factor, and highway transportation plays a key role in influencing logistics energy consumption and carbon emissions indexes. The findings can provide valuable references and methodologies, as well as support for decision-making in the sustainable development of logistics system.

## Introduction

In the new era, the increasing diversity and high-quality logistics demand adds more complexity to the existing logistics system as the consumers’ demand and purchase behavior are changing. The solution to this challenge is to improve the coordination among endogenous, exogenous, and symbiosis dynamics factors within the logistics system, and to achieve sustainability and economic growth [1]. However, the increasingly severe threat of internal and external conditions have affected the sustainability of logistics system, the local economy and the environment. Although some mandatory actions are believed to be effective in alleviating problems, an unprecedented incident has severely limited the potential service of logistics system.

Furthermore, the negative externalities of logistics transportation freights of heavy-duty vehicles have deemed as one of major source of carbon emissions [2]. It is against the requirement of the sustainability of logistics system. which is defined based on the relevant reviews and logistics development strategies. It starts from the functional element, supporting element, flow element, logistics subject and network element of system, and constructs an organic aggregate of multiple logistics units for safeguarding people’s need, and forms a symbiotic logistics ecosystem for social-economy, and environment. Actually, reducing carbon emissions from logistics sector is conducive to alleviate its intensity to 60%-65% from 2005 to 2030, which proposed by China at the Paris Climate Conference [3].

To deal with the prevailing issues for non-sustainability, understanding of the integrated framework of logistics system is of crucial importance in establishing and maintaining sustainable development. Chen et al. [4] analyzed the development of the port logistics system for outbreak emergency supplies from the overseas. Jiang et al. [5] developed a thorough and systematic evaluating index of emergency logistics system reliability for epidemic prevention. Although the existing literature provides an in-depth understanding of sustainability, a “dynamic” system with complicated elements and relations remains unexplored. It is thus a challenge to comprehensively analyze the combined effects of various indexes on logistics system [3], and open up the inner mechanism of the sustainability of logistics system.

The system dynamics (SD) method, which provides a better understanding of the complex system problems in reality, is well suited to test the complex phenomenon that occurs within logistics system. More specifically, as a simulation technique, the SD approach provides a convenient means for resting scenarios involving various factors and uncertainties. For example, Tian and Li [6] established a system dynamics model to illustrate the quantitative relationship between the green finance system and CO_2_ emissions. Fontoura et al. [7] used the approach of system dynamics to examine the influence of Brazilian policies on urban transportation system. Despite the fact that scholars have studied the relevant issues of logistics system, and the SD method has made great progress, these studies are rather isolated, and do not take a systematic perspective on the interrelationships among logistics factors or consider its prospects.

Overall, a limited amount of research has been conducted the key mechanism that affect logistics system, area economic and environment. The novelties of this study are elaborated: Firstly, we design the structure of logistics system from a systematic perspective, and characterize a series of key factors that have an impact on logistics system, the local economy and environment. Secondly, since the SD approach can well simulate the non-linear dynamics, system feedback, behavioral response, and alternative scenarios [8], we dynamically discern the process of logistics system with the SD model. Thirdly, we conduct quantitative research with the statistical data by Vensim PLE software. On the basis of verifying the validity and rationality of model, we set multiple scenarios, namely adjustment of the system input, transportation mode, and the industrial structure scenarios. Finally, some strategies, conclusions and future research are proposed for facilitating the sustainability of logistics system.

The remainder of this study is organized as follows. The recent literature reviews which concern logistics system and methodology are described in Section 2. Section 3 designs the SD model, which covers system boundaries defining, causal loop diagram analyzing, and stock-flow mapping. Followed by the validation of system modeling, results of extending numerical scenarios are presented in Section 4. Section 5 draws conclusion, and offers the potential line for future investigation.

### Literature review

Two branches of the literature reviews are related as follow: the structural analysis of logistics system, and the system dynamics methodology which has been applied broadly and extensively.

### Structure analysis of logistics system

Logistics system is defined on a micro-scale as an organized system that performs specific functions within a given area [9], which usually incorporates the sophisticated interactions and various feedback between the social, logistics, and economic factors. Although early studies have proved the effect of the organization and coordination, carbon emissions, logistics information and transportation, policy system on logistics processing [4, 10], but they are weak in revealing the complexity of the structure analysis of logistics system. Based on the relevant reviews and system elements (e.g., logistics functional and supporting elements, logistics subjects, etc.), logistics system can be divided into four main parts: logistics transportation and distribution, logistics information management, organization command and coordination, and reverse logistics management.

As a central component of logistics system, it is possible to employ logistics transportation and distribution for improving system performance, and enhancing the rationality of terminal logistics activities [11]. Logistics information management plays an essential role in monitoring real-time logistics dynamics and ensuring that the system is running smoothly. There are many applications for the warehouse information management, such as the enterprise warehouse planning system, the warehouse management system, and the warehouse control system [11]. Similarly, studies have focused on technological innovations. Cruz-Jesus et al. [12] developed a model using the framework of technology-organization-environment. Relative advantages and compatibility of technology were also analyzed by Yoon [13].

Organization command and coordination is vital in the logistics system, which not only shoulders a crucial role in integrating logistics resource, but also has an inseparable relationship with logistics information management [14]. A new method, which influences the environmental uncertainty in logistics outsourcing relationship, was proposed by Yang and Zhao [15]. With an increasing threat to the system, it is crucial for logistics organizations to shoulder the social responsibility. Sennaroglu and Celebi [16] observed that a significant organization can constantly adjust its behavior to cope with logistics problems.

Recovering valuable materials that exist in the circulation of logistics system is gradually recognized as a critical issue, along with globally emerging environmental awareness and mandatory acts [17, 18]. The definition of reverse logistics was firstly proposed by Stock [19]. Later, Rogers and Tibben-Lembke [20] offered its developed definition, which involves the process of planning, implementing, and controlling the effective flow of materials. From 2001 to 2014, its research mainly covered general studies, waste management, recycling, and disassembling [21]. Sung et al. [22] discussed the problem of how to efficiently utilize the sensor data and IoT technology in reverse logistics. Furthermore, the motivations of sustainable reverse logistics focusing on environmental protection have been revealed [23, 24]. Ho et al. [24] pointed out that reverse logistics can help achieve economic and environmental goals.

### System dynamics methodology

As a method for exploring the complex connections between subsystems and their intricate effects, a system dynamics (SD) method is acknowledged as a powerful approach to deal with linear and non-linear interactions [25]. Which was initially developed by the Massachusetts Institute of Technology (MIT) Sloan School of Management and the MIT System Dynamics Group in the 1960s. In comparison to other regression analysis methods, the SD method has the advantage of studying that is inherent to systems within a long-term dynamic process [26]. At the same time, scholars have expanded the SD modeling towards micro-analytic models for various logistics issues. Hennies et al. [27] applied a mesoscopic SD approach that combined discrete events and continuous flows in a hybrid DES-SD model.

A summary of applying system dynamics is presented in Table 1, it can be noted that there are many possibilities of research to apply the SD approach to various fields, such as the transportation [7], business [28] and the administration sector [29]. Cao et al. [30] developed the SD model to simulate the scenarios on CO_2_ emission mitigation in the whole life cycle of the green electric-coal supply chain. Research has also been conducted with regard to SD approach for urban development. Dong et al. [31] employed a SD method to examine the relationship between the implementation strategy of underground logistics system (ULS) and the sustainability of urban transportation and logistics.

**Table 1:** Summary of applying themes of system dynamics.

Reference	Year	Aim of study	Methodology	Theme(s)
Noto and Bianchi (2015)	2015	To understand the impact of the governing structure on system’s performance	SD model	Administration sector
Cosenz et al., (2019)	2019	To examine social, environmental and economic drivers of a company	SD model	Business sector
Cao et al., (2019)	2019	To analyze the life cycle of supply chain with greening energy intensive indicator	SD model	Electrical supply chain
Dong et al., (2019)	2019	To discuss the impact of underground logistics system on urban sustainable development	SD approach	Urban development
Fontoura et al., (2019)	2019	To analyze the impact of urban transportation policy	SD approach	Transportation sector

Based on the above literature, the contribution of this article can be identified: First, unlike most of the studies that focus on a single process, this study regards logistics system as a whole. A series of key factors for system operations and the external impacts are incorporated, such as the logistics transportation and distribution, organization command and coordination, logistics information management, and especially reverse logistics management. Second, although the SD method has been widely used, only a small percentage of them considers the carbon emissions and reverse logistics management factors. This study investigates the relationship between the economy-operations subsystem, carbon emission subsystem, and logistics service subsystem with the SD model. Third, while several reviews have explored the issues about logistics system, no specific studies have been conducted on the amplitudes of logistics variation rates with the statistical data obtained from different scenarios. We investigate several scenarios to detect the effect of adjusting the system input, transportation mode, and the industrial structure. The findings contribute to simulating the logistics scenarios and providing valuable insights.

### Model design and analysis

Modeling on the integrated scheduling of logistics system that recognizes the interdependence for eco-friendly logistics system is necessary. But it is still unclear how a logistics system model can be constructed to evaluate system performance from the systematic perspective. On the basis of the characteristics and structure of the logistics system, a system dynamics approach is applied, which explicitly aims to facilitate the understanding of such complex systems and the construction of models that describe their characteristics [6]. This section follows the three stages of consisting SD model, which are identifying the system boundaries, designing the causal loop diagram, and establishing the stock-flow diagram [32].

### System boundaries defining

Logistics system, as an important part of the social-economy, has an inextricably interdependent connection with regional economy. The growth of economic benefit is contributed to improve the service capacity of logistics system, through the investment in logistics technology and equipment. Wang et al. [33] illustrated an IoT-based intelligent logistics dispatching system. A macroscopic system which consists three states such as transportation, activity, and environment systems was discussed by Maheshwari [34]. Besides, logistics transportation could cause a rapid increase in logistics energy consumption, resulting in carbon emissions and pollution loss, and increasing the difficulty of the sustainability of logistics system and economy-operations [25, 30, 35]. Wang et al. [25] proposed a complex system model to simulate the impact of jobs-housing relationship adjustment policies on CO_2_ emissions from urban transport. It is therefore necessary to explore the systemic interactions between regional economy, carbon emissions, and logistics system, which is rarely explored by existing studies.

**Fig 1:**
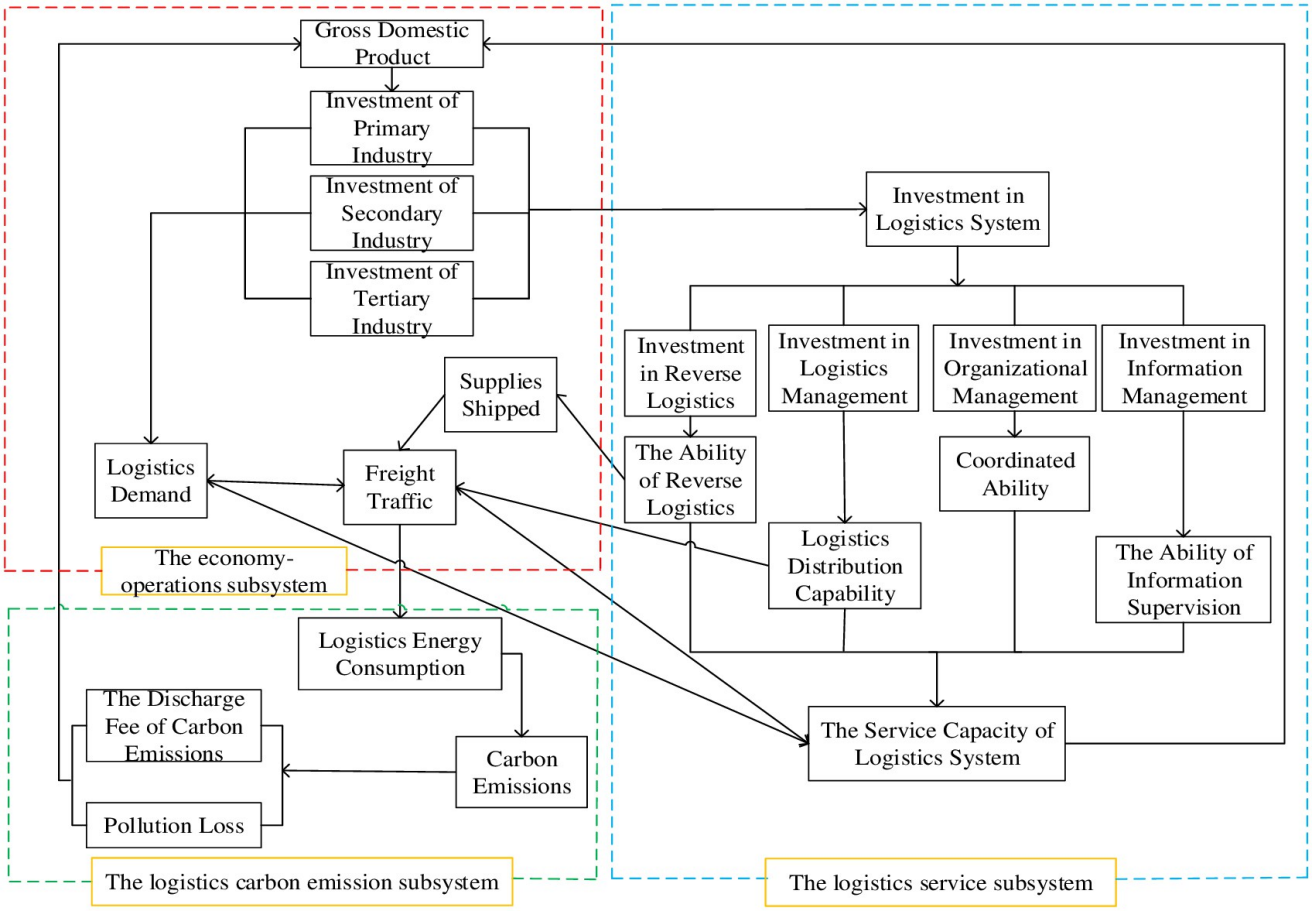
Functional relationship of major subsystems. Source: Authors’ own work.

To understand the mutual interactions and influences among main factors, this study considers that the SD modeling is adequately reflected in the chosen boundaries. As shown in Fig 1, it can be divided into three parts: the economy-operations subsystem, the logistics carbon emission subsystem, and the logistics service subsystem. Each subsystem is indicated by the dotted line with different colors, and the mutual effects and interactions of indicators are presented by the arrows.

In the economy-operations subsystem, GDP mediates the investment of primary, secondary, and tertiary industry, and then determines the value of investment in logistics system. The economy field’s improvement in freight traffic that links to the supplies shipped and logistics demand parameters will increase the logistics energy consumption, resulting in the discharge fee of carbon emissions and pollution loss which can affect the value of GDP factor. There is a nonlinear relationship between logistics demand and GDP, and the system service capacity has a positive impact on economic growth. Meanwhile, it studies the reaction force of changes in the economy-operations subsystem to other subsystems under the established premise of economics, logistics, and carbon emission.

In the logistics service subsystem, the level of system investment is incorporated into a dynamic system model, which influences the ability of reverse logistics, logistics distribution, information supervision, and coordinated organization. The service capacity of logistics system, which is a factor of positive feedback on GDP, has an impact on the logistics demand and freight traffic. Following the inevitable increase in logistics supply capacity, the improvement of logistics system will create the condition for further economic growth.

Carbon policy support and restraint mechanism of the logistics carbon emission subsystem performs a balanced function in the overall logistics system, and this subsystem is regulated by constraint index and support index changes. Logistics transportation sector is considered highly responsible for its deleterious impact on air quality, as it has a side effect on the logistics energy consumption that influences the value of carbon emissions. It is noted that this factor has directly affected the discharge fee of carbon emissions and pollution loss factors, which have gradually put a strain on GDP. Table 2 depicts some selected factors that are employed to create the SD model. It is worth noting that other factors and abbreviations of this model are illustrated in Table A (seen S1 Appendix for more details).

**Table 2:** Main factors and abbreviations of SD model.

Categories	Relative factors	Abbreviation	Sources
Economy-operations subsystem	gross domestic product	GDP	Shen et al., (2019); Jayathilaka et al., (2022)
	primary industry	PI	Beijing Statistical Year Book (2010–2019)+
	secondary industry	SI	Beijing Statistical Year Book (2010–2019)
	tertiary industry	TI	Beijing Statistical Year Book (2010–2019)
	logistics demand	LD	Jacyna (2013); Jiang et al., (2020)
	freight traffic	FT	Amaya et al. (2021); Marcucci et al., (2017)
Logisticscarbon emission subsystem	logistics energy consumption	LEC	Ye et al., (2021); Wang et al., (2021)
	pollution loss	PL	Masoumik (2015); Yang and Zhao (2016)
	the discharge fee of carbon emissions	CEDF	Beijing Master plan (2016–2035) and Beijing Urban Traffic Annual Report (2010–2019)
	carbon emissions	CE	Lu and Sun (2020); Tian and Li (2022)
	the consumption of highway transportation	HTC	Beijing Master plan (2016–2035) and Beijing Urban Traffic Annual Report (2010–2019)
	the consumption of railway transportation	RTC	Beijing Master plan (2016–2035) and Beijing Urban Traffic Annual Report (2010–2019)
	the consumption of civil aviation transportation	CATC	Beijing Master plan (2016–2035) and Beijing Urban Traffic Annual Report (2010–2019)
	the consumption of pipeline transportation	PTC	Beijing Master plan (2016–2035) and Beijing Urban Traffic Annual Report (2010–2019)
Logistics service subsystem	investment in logistics system	LSI	Jiang et al., (2020); Jayathilaka et al., (2022)
	logistics facility input	LFI	Marcucci et al., (2017); Jayathilaka et al., (2022)
	investment in logistics talent training	LTTI	Beijing Statistical Year Book (2010–2019)
	investment in advanced technology	ATI	Zhang et al., (2021); Lu and Sun (2020); Kembro et al., (2017)
	the equipment investment of reverse logistics	RLEI	Rogers and Tibben-Lembke (1999); Sung et al., (2020)
	organizational communication ability	OCA	Liu et al., (2020); Zheng et al., (2021)
	decision-making ability	DMA	Kembro et al., (2018); Yan et al., (2022)
	organization system	OS	Jiang et al., (2020); Zheng et al., (2021)
	organizational guarantee system	OGS	Zhu and Kraemer (2005); Jiang et al., (2020); Fontoura et al., (2019)
	research expenditures	RE	Zhang et al., (2021)
	logistics distribution capability	LDC	Dong et al., (2019); Jayathilaka et al., (2022)
	information supervision ability	ISA	Liu et al., (2020)
	the ability of reverse logistics	RLA	Jian et al., (2019); Sung et al., (2020)
	coordinated ability	CA	Kembro et al., (2018); Zheng et al., (2021)
	the service capacity of logistics system	LSSC	Dong et al., (2019); Liu et al., (2020)

### Causal loop diagram

There are complex inter-relationships between internal factors that influence and/or restrict each other [25], it is essential to reveal the mechanism of logistics system through the economic, societal, and environmental aspects. After the boundary clarification process, this section develops the causal loop diagram (CLD), which is a useful tool for demonstrating the feedback structure of system elements, and indicating which factors in the dynamic system cause a change in the other.

**Fig 2:**
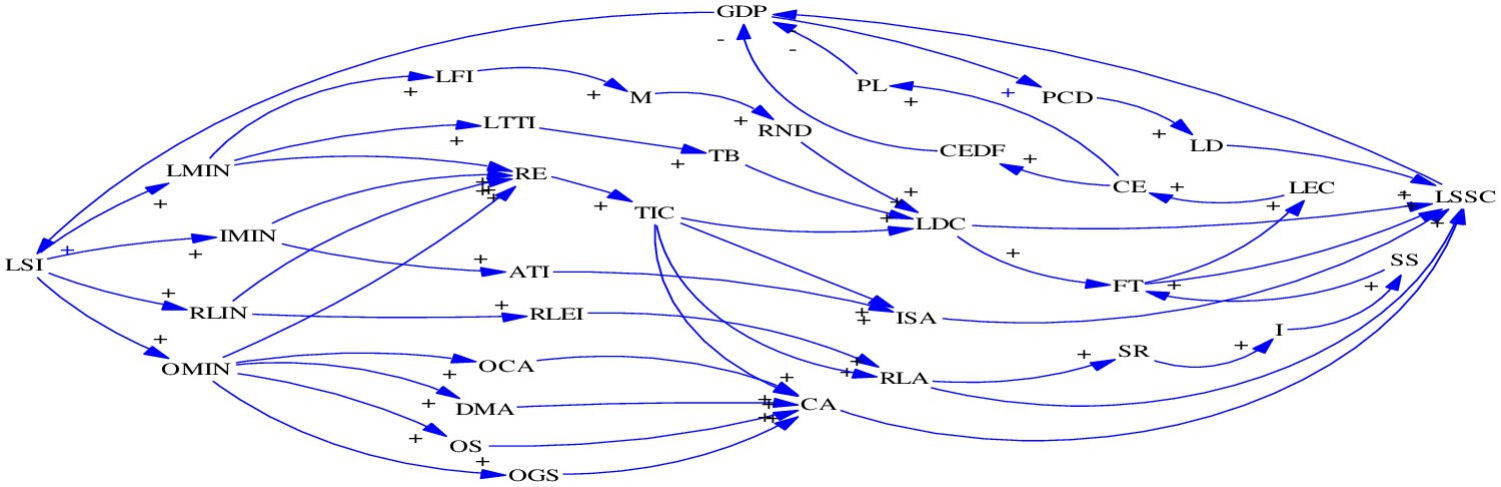
Causal loop diagram. Source: Authors’ illustration based on literature.

Based on the identification of causal connections among factors, we employ the SD method to illuminate the trade-off between system indicators and bidirectional causes. The CLD of system model is illustrated in Fig 2. The main factors of feedback loops are connected by arrows. Each arrow, which represents a causal relationship between two factors, is marked with a polarity to indicate the positive or negative influence. The positive polarity means that two factors will change in the same direction, and the negative polarity means the reverse [31]. Moreover, due to the limited space, the fourteen feedback loops in the CLD are shown in Table 3, which includes six reinforcing loops and eight balancing loops.

**Table 3:** Main feedback loops in the CLD.

Category	Causal feedback loops
Reinforcing loop 1	GDP→+LSI→+LMIN→+LFI→+M→+RND→+LDC→+LSSC→+GDP
Reinforcing loop 2	GDP→+LSI→+LMIN→+LTTI→+TB→+LDC→+LSSC→+GDP
Reinforcing loop 3	GDP→+LSI→+IMIN→+RE→+TIC→+ISA→+LSSC→+GDP
Reinforcing loop 4	GDP→+LSI→+IMIN→+RE→+TIC→+RLA→+LSSC→+GDP
Reinforcing loop 5	GDP→+LSI→+OMIN→+OCA→+CA→+LSSC+GDP
Reinforcing loop 6	GDP→+LSI→+OMIN→+OGS→+CA→+LSSC+GDP
Reinforcing loop 7	GDP→+ISI→+RLIN→+RLEI→+RLA→+LSSC→+GDP
Reinforcing loop 8	GDP→+PCD→+LD→-LSSC→-GDP
Balancing loop 1	GDP→+LSI→+RLIN→+RE→+TIC→+RLA→+SR→+I→+SS→+FT→+LEC→+CE→+CEDF→-GDP
Balancing loop 2	GDP→+LSI→+RLIN→+RE→+TIC→+RLA→+SR→+I→+SS→+FT→+LEC→+CE→+PL→-GDP
Balancing loop 3	GDP→+LSI→+LMIN→+LFI→+M→+RND→+LDC→+FT→+LEC→+CE→+PL→-GDP
Balancing loop 4	GDP→+LSI→+LMIN→+LTTI→+TB→+LDC→+FT→+LEC→+CE→+PL→-GDP
Balancing loop 5	GDP→+LSI→+IMIN→+RE→+TIC→+RLA→+SR→+I→+SS→+FT→+LEC→+CE→+PL→-GDP
Balancing loop 6	GDP→+LSI→+IMIN→+RE→+TIC→+RLA→+SR→+I→+SS→+FT→+LEC→+CE→+CEDF→-GDP

Reinforcing loop 1 shows that GDP plays an important role in increasing the investment in logistics system (LSI), which is conducive to improving the value of logistics management. Some measures are taken to enhance the performance of logistics distribution, such as increasing the logistics facility input and improving the density of road network, which can bring positive feedback to the service capacity of logistics system (LSSC). Ultimately, the value of GDP will be improved.

Reinforcing loop 7 indicates that GDP has an influence on the investment in reverse logistics (RLIN) through increasing the system investment. Moreover, RLIN effects both the equipment investment of reverse logistics (RLEI) and the ability of reverse logistics (RLA), to a certain extent, which will increase the coordinated development of LSSC and GDP.

Reinforcing loop 8 reveals that GDP reflects the situation of logistics demand (LD) by influencing the value of per capita demand (PCD). The behavior is represented by the LD and LSSC factors with a negative sign. It is attributed to the fact that a surging logistics demand may be unfulfilled. In general, a decrease in the amount of LD leads to a further negative change in the LSSC and GDP. It is noted that satisfying the actual logistics demand is helpful to enhance the system service capability.

Balancing loop 1 demonstrates that there will be a growth of RLIN when the LSI increase by GDP. The higher RLIN, the more it can give rise to the growth of research expenditures. While it will simulate the supplies recycled (SR) parameter to increase the inventory of recyclable materials, and the volume of freight traffic (FT). However, the FT increase will finally affect the value of logistics energy consumption (LEC) and carbon emissions (CE), and have the negative impact on GDP.

Balancing loop 4 is a representation of the positive feedback between GDP and the investment in logistics management (LMIN), which is a more effective approach to enhance the training for logistics talent. The higher LDC, the better it can improve the value of FT that results in the increase of LEC and CE in the overall system process. In addition, the increasing pollution loss (PL) factor seriously affects the efficiency of GDP.

Balancing loop 5 expresses that LSI is affected by GDP, and contributes to improving the performance of information management, with a positive correlation among the RE and TIC factors. Enhancement in the ability of reverse logistics is an effective strategy in influencing both the inventory of recyclable materials and the supplies recycled factors. However, the improvement of FT will inevitably lead to the increase of LEC and CE, and cause the output of PL to have a counteractive impact on GDP.

### Stocks and flows

**Fig 3:**
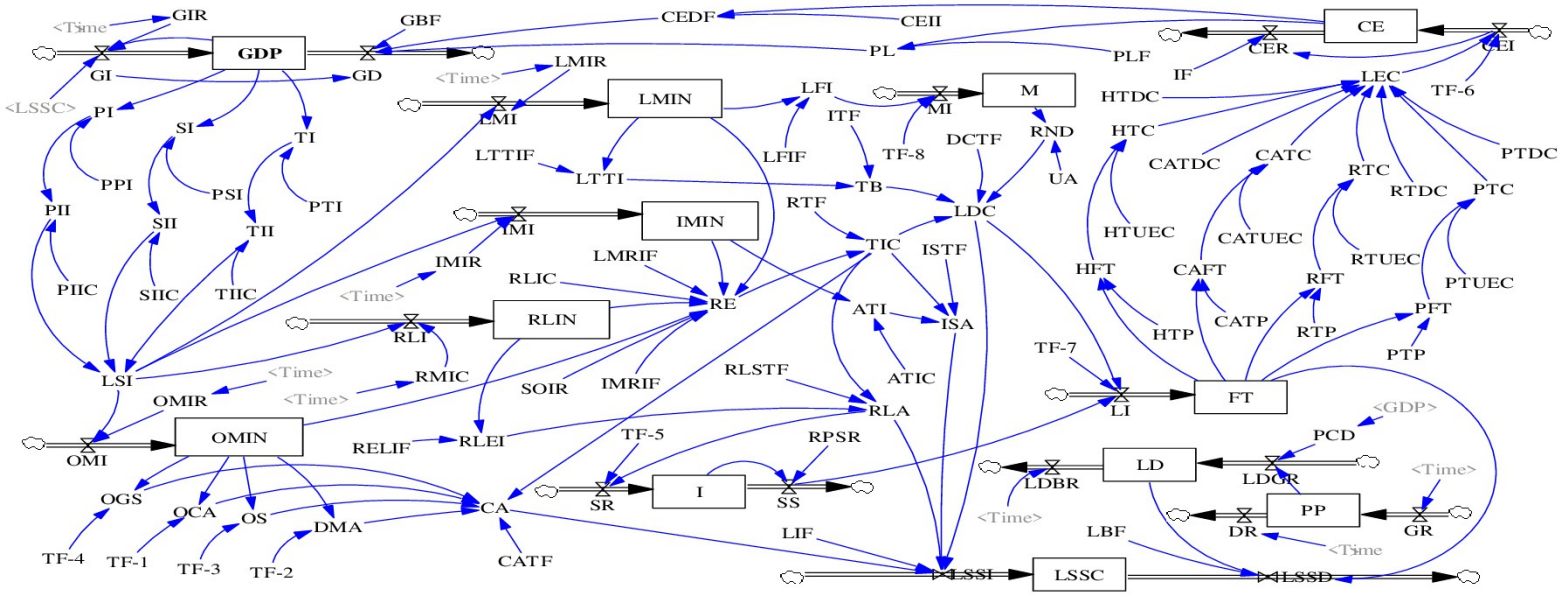
Stock and flow diagram. Source: Authors’ own work.

To simulate this conceptualized model, the next stage is to move from the qualitative study of the causal loop diagram to a quantitative model, which is the most crucial step in system dynamics. As illustrated in Fig 3, these factors in the stock and flow diagram are divided into the 12 level variables, 18 flow variables, and 95 auxiliary variables. Similarly, through the availability of data and structural equilibrium among indicators, the main factors and formulas of stock-flow mapping can be shown in Table 4.

**Table 4:** Main factors and equations of stock-flow mapping.

Categories	Factors	Equations	Categories	Factors	Equations
Economy-operations subsystem	GDP	INTEG (GI-GD, Initial value)	Logistics servicesubsystem	LSI	PII+TII+SII
	PI	GDP*PPI		ATI	IMIN*ATIC
	SI	GDP*PSI		RLEI	RELIF*RLIN
	TI	GDP*PTI		OCA	OMIN*"TF-1"
	LD	INTEG (LD*(LDGR-LDBR, Initial value)		RE	IMRIF*IMIN+LMIN*LMRIF+OMIN*SOIR+RLIN*RLIC
	FT	INTEG (LI, Initial value)		LSSC	INTEG (LSSI-LSSD, Initial value)
Carbon emission subsystem	CE	INTEG (CEI-CER, Initial value)		CA	(TIC+OGS+DMA+OCA+OS)*CATF
	PL	CE*PLF		DMA	"TF-2"*OMIN
	CEDF	CE*CEII		LFI	LMIN*LFIF
	HTC	HFT*HTUEC		LTTI	LMIN*LTTIF
	RTC	RTUEC*RFT		LDC	(TIC+TB+RND)*DCTF
	CATC	CATUEC*CAFT		ISA	TIC+ISTF*ATI
	PTC	PTUEC*PFT		RLA	(RLEI+TIC)*RLSTF

Fig 3 presents three subsystems, namely, the economy-operations subsystem, the logistics carbon emission subsystem, and the logistics service subsystem. During the design of a mathematical model, this article deduces the quantitative equations of logistics factors and the non-linear dynamics relationship into subsystems.

(1) Economy-operations subsystem, which specifies the traffic volume of freight and the per-capita disposable income of individuals, is the most important factor in increasing the total logistics demand. GDP is calculated by Eq (1), in which INTEG stands for the function of integral [31]. Eq (2) is helpful to analyze the logistics demand (LD), in which LDGR represents the growth rate of LD, and LDBR illustrates the baffle rate of LD. The freight traffic [36, 37] stock is the function of logistics increase, as shown in Eq (3).


GDP=INTEG(GI-GD,Initialvalue) (1)

LD=INTEG(LD*(LDGR-LDBR,Initialvalue) (2)

FT=INTEG(LI,Initialvalue) (3)

(2) Logistics carbon emission subsystem depicts several factors that have a direct or indirect effect on GDP. In general, if the tolerated threshold of the freight volume and emissions is exceed, it will cause the socioeconomic losses. As illustrated in Eq (4), the carbon emissions factor [38, 39] is the function of the increase of carbon emissions (CEI) and the decrease of carbon emissions (CER) value. By varying the standard of CE, PLF, and CEII factors, the results of the discharge fee of carbon emissions (CEDF) and pollution loss (PL) parameters change accordingly. It considers the CEDF and PL as factors to analyze that the impact of the CE on GDP, as shown in Eqs (5) and (6).


CE=INTEG((CEI-CER,Initialvalue) (4)

$$\text{PL}={\text{CE}}^{*}\text{PLF}$$ (5)

$$\text{CEDF}={\text{CE}}^{*}\text{CEII}$$ (6)

(3) As previously stated, the service capacity of logistics system (LSSC) and the investment in logistics system (LSI) are the main factors within the logistics service subsystem. Based on the related literature and expert guidance, LSI relies heavily on the investment of primary, secondary, and tertiary industry. As presented in Fig 3, the coordinated ability (CA) depends on the organization system, technology innovation, organizational guarantee, decision-making, and organizational communication ability. In Eq (9), the technological innovation, network density, and talent benefit factors are selected to present the distribution capability of logistics. Eq (10) illustrates a calculation method of the ability of information supervision (ISA). As seen in Eq (11), the ability of reverse logistics (RLA) is affected by the innovation capability and equipment investment of reverse logistics. The function of the LSSI and LSSD factor is stated in Eq (12).


LSI=PII+TII+SII (7)

$$\text{CA}={\left(\text{TIC}+\text{OGS}+\text{DMA}+\text{OCA}+\text{OS}\right)}^{*}\text{CATF}$$ (8)

$$\text{LDC}={\left(\text{TIC}+\text{TB}+\text{RND}\right)}^{*}\text{DCTF}$$ (9)

$$\text{ISA}=\text{TIC}+{\text{ISTF}}^{*}\text{ATI}$$ (10)

$$\text{RLA}={\left(\text{RLEI}+\text{TIC}\right)}^{*}\text{RLSTF}$$ (11)

LSSC=INTEG(LSSI-LSSD,Initialvalue) (12)

## Model validation and simulation

As the capital of China, Beijing is selected as a study example for the following reasons: First, with accelerated urbanization, changes in the urban functional layout have occurred in recent years, leading to separation of jobs-housing and spatial dislocation phenomena in Beijing [25]. Second, Beijing’s logistics system presents some features of the diversified logistics subjects, the “three rings, five belts and multi-centers” of logistics spatial pattern, and the dominant position of terminal logistics. In addition, with the consideration of the pandemic, it’s crucial to simulate the current logistics system of Beijing.

Since a SD model requires real data to verify its effectiveness, the modeling time horizon of logistics system for the evolution in Beijing is set to 13 years from 2010 to 2022, this paper sets the step size as one year and the model period as 2010. Table 5 lists the detailed values and reference sources for the main factors of model. Most of them are sourced from Beijing Statistical Year Book (2010–2019) [40], Beijing Master plan (2016–2035) and Beijing Urban Traffic Annual Report (2010–2019). Additionally, some parameters of the model are defined by the expert consultation, historical and actual trends, linear regression, and curve fitting methods. The data is analyzed with Vensim PLE software, which combines model building, systems thinking, and variable analysis techniques.

**Table 5:** Values of main factors in the simulation.

Acronyms	Initial value	Unit	Source	Acronyms	Value	Unit	Source
GDP	14964	100 million yuan	Gov.document	RPSR	0.87	Dmnl	Assumed
LD	5.13658e+06	10000 ton-km	Gov.document	RLIC	0.2	Dmnl	Assumed
CE	3068	10000 tons	Gov.document	GBF	9.8e-08	Dmnl	Assumed
PP	1961.9	10000 persons	Gov.document	PLF	0.015	Dmnl	Assumed
LSSC	0	Dmnl	Assumed	HTP	0.555	Dmnl	Assumed
FT	4.48157e+06	10000 ton-km	Gov.document	RTF	0.118	Dmnl	Assumed

### Model validation

In purpose of validating the logistics system model, this section has not only presented the running test of model, but also displayed the stability, sensitivity and the historicity test of model.

### Running test of model

Its known that the running test includes the examination of model structure and variables units. To check the rationality of the constructed model, we applied the simulated soft to test its structure. “Model is OK” can be illustrated, which presents the structural consistency of model. Moreover, the units check tool is provided to analyze the magnitudes of factors. After several compilation error and follow-up checks, the model is finally made to verify the consistency of dimension, and the correctness of equation.

### The stability of model

**Fig 4:**
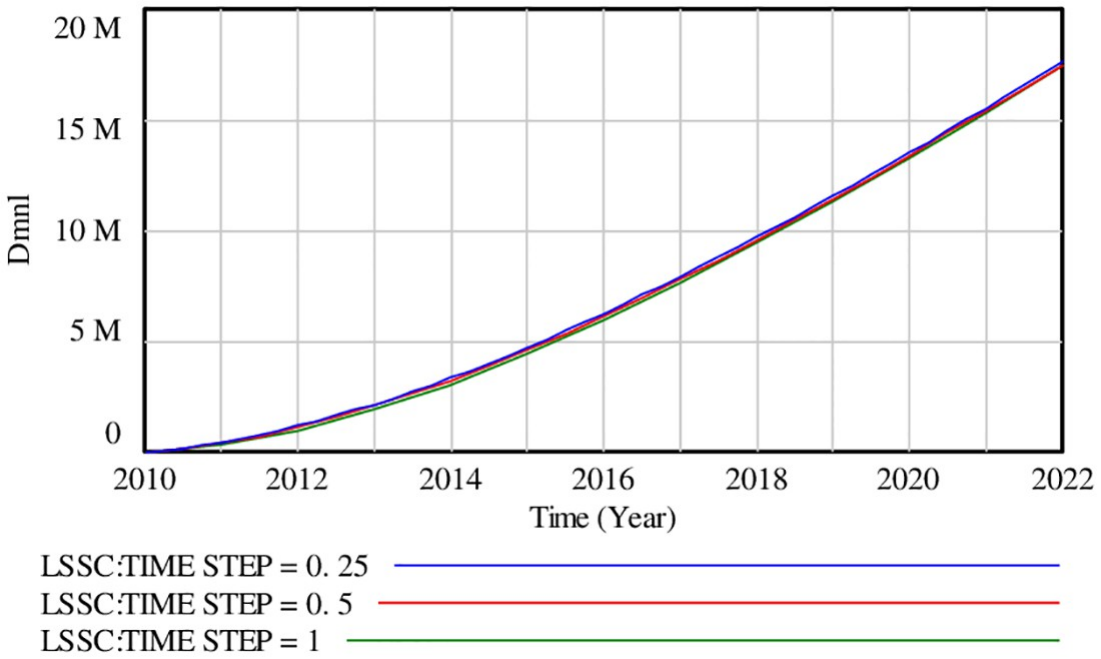
Changes in the stability of model triggered by the TIME STEP.

The next step is to examine the stability of logistics system model. If the value of factors vary significantly under the different time intervals, it means that system is not scientifically stable. Through the method of integral error test, the service capacity of logistics system is investigated in Fig 4. It illustrates that the curve of LSSC is basically consistent from 2010 to 2022, and the model has a good stability. Similarly, other indicators within model also passed this test.

### The sensitivity of model

**Fig 5:**
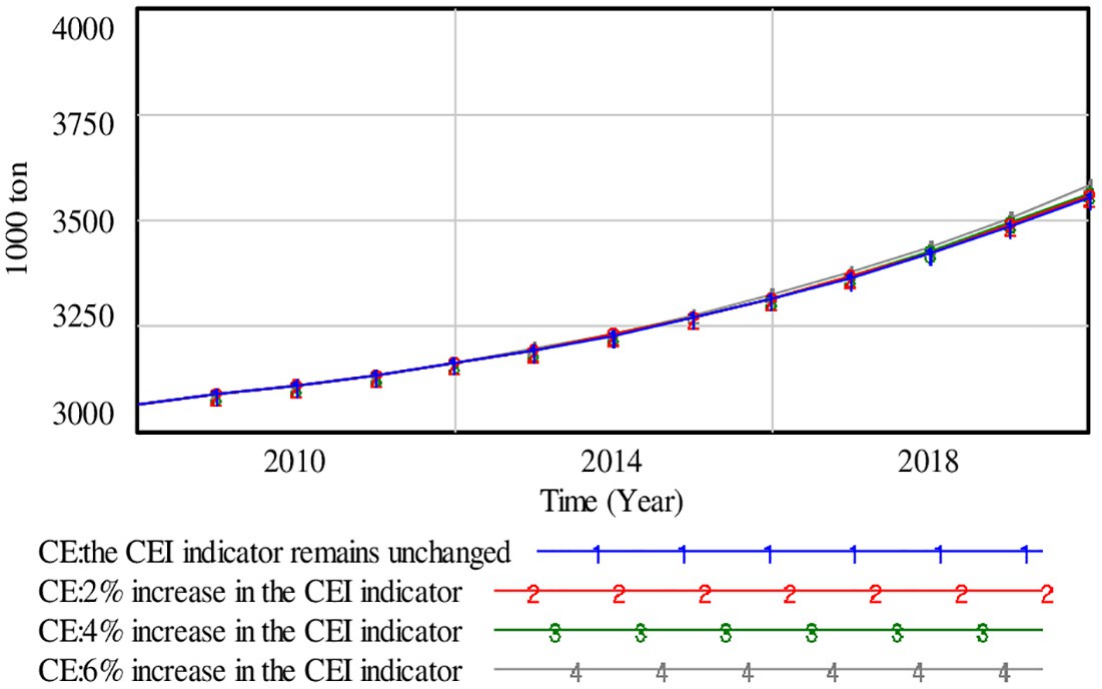
Changes in the sensitivity of model triggered by the CEI indicators.

The sensitivity analysis focuses on checking the model’s response to changes in input parameters. Taking the CE factor as the example, the simulated result of CE indicator through adjusting the rate of CEI is demonstrated in Fig 5. A change in one parameter does not have a significant effect on the result of model, and the sensitivity of model is good.

### Historicity test of model

Taking into account the mandatory frequency of a larger number of factors, the GDP and logistics demand factors in model are selected. This study regards GDP as the level of economic development, and expresses logistics demand as the cargo turnover. Table 6 shows the error percentage of the simulated and actual value of testing factors. Results show that the error range of GDP from 2010 to 2019 is -6%-0.2%, with an average error of -2.0380%. The error range of logistics demand factor is between -0.02% and 0.07%, an average error of 0.00482%, and the error percentage less than 6%. The high similarity between the simulation values of the real values and model implies that the behavior described by SD model is well consistent with the actual state, and then proves the confidence and validity of modelling.

**Table 6:** Results of the historical and simulated values.

Year	GDP/100 million yuan			Logistics demand/10000 ton-km		
	Real value	Simulated value	Error/%	Real value	Simulated value	Error/%
2010	14964.00	14964.00	0.0000	5136580	5136580	0.00000
2011	17188.80	16947.80	-1.4021	6169272	6169030	-0.00392
2012	19024.70	19060.30	0.1871	6383052	6383060	0.00013
2013	21134.60	20818.30	-1.4966	6809063	6809000	-0.00093
2014	22926.00	22813.70	-0.4898	6728238	6728610	0.00553
2015	24779.10	24558.10	-0.8919	6236947	6236120	-0.01326
2016	27041.20	26409.20	-2.3372	6713288	6713180	-0.00161
2017	29883.00	28534.70	-4.5119	7000530	7005200	0.06671
2018	33106.00	31162.20	-5.8714	7806542	7806600	0.00074
2019	35371.30	34110.00	-3.5659	9007714	9007250	-0.00515
Average error/%			-2.0380	Average error/%		0.00482

## Results and discussion

Considering the advantages of SD approach, such as the inclusion of external logistics factors, and the limited data, this study first tests the tendency of economic and logistics development in Beijing, and then simulates the behavior mode of system under different scenarios. Some high-leverage solutions are proposed to improve the service level of system, with more attention to the coordinated area growth.

### Model simulation and prediction

**Fig 6:**
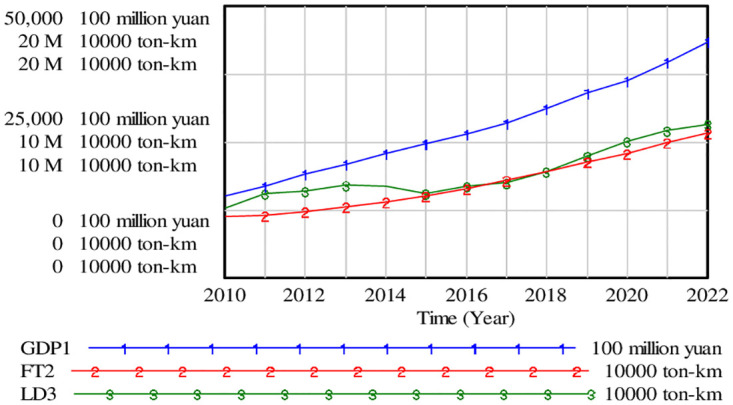
Simulation trend chart of economy and logistics development.

Fig 6 indicates that the tendency of economic and logistics development in Beijing, where the economy is showing a steady upward trend. As a result of the new era of urban orientation, Beijing strictly follows the features of logistics system, and plays the two-way guiding role of market and system with the help of complementary industrial policies. The carve FT2 and LD3 reveal the difference in the slowly rising trend of LD, while FT illustrates a first decline and then a slow growth trend. From 2010 to 2014, logistics demand was higher, with the largest gap in 2012. Since customer needs are continuously changing, along with the expansion of e-commerce, the logistics industry needs to combine a variety of channels to support the seamless shopping experiences [41, 42]. However, practical factors such as insufficient investment in logistics equipment and technologies may hinder the growth of FT factor. From 2015 to 2018, the FT factor in Beijing satisfied the actual demand. It reveals that the logistics industry has gradually paid attention to logistics innovation. Its also related to the fact that LD itself has declined. From 2019 to 2022, capital logistics supply and demand will fork again, and that gap will be narrowed. It is expected that the new intersection point will appear around 2024, which is inseparable from the painful experience of pneumonia forcing logistics to use green technologies or products.

### Scenarios design and analysis

To accurately comprehend the model and policy adjustment of Beijing, several scenarios of different types are designed, which are the industrial structure adjustment, the transportation mode, and the system input, respectively. At the same time, some incentive strategies are highlighted based on the effect of those scenarios on logistics performance.

### (1) Adjustment of industrial structure

**Fig 7:**
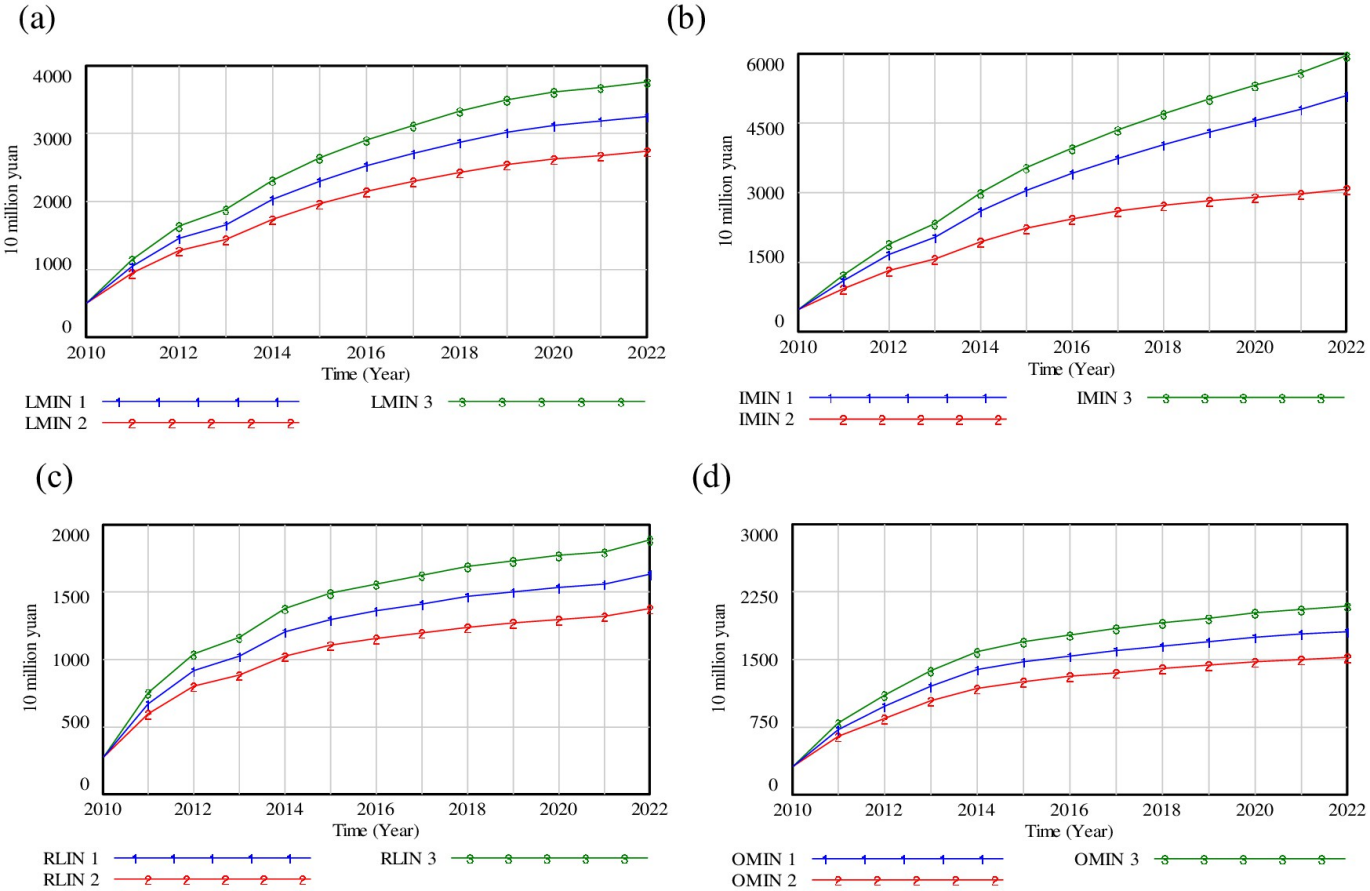
Simulation results of adjusting of industrial structure. (a) LMIN; (b) IMIN; (c) RLIN; (d) OMIN.

To understand the impact of adjusting industrial structure on system service, three scenarios are established, namely, scenario (1) is the original condition; scenario (2) demonstrates that the input coefficient of tertiary industry is increased by 8%, while the input coefficient of primary industry and secondary industry is decreased by 4%; scenario (3) represents that the input coefficient of tertiary industry is decreased by 8%, while the input coefficient of primary industry and secondary industry is increased by 4% respectively. The simulation results are illustrated in Fig 7.

Fig 7(a) shows that the growth rate of LMIN in scenarios is relatively slow from 2010 to 2013, but since 2014, the rate rises again. Fig 7(b) depicts that IMIN 2 will decrease by 39.81% compared with the base scenario by 2022, while the value of IMIN 3 will increase by16.98% compared with the curve of IMIN 1 in 2022. Fig 7(c) shows that the level of RLIN 2 had risen to 799.933, 884.473, 1028.11 (10 million yuan) by the years of 2012, 2013 and 2014. The result of RLIN 3 is about 1.137 times that of RLIN 2 in 2022. As shown in Fig 7(d), by 2022, the OMIN 3 will increase to 2092.38 (10 million yuan), which has a growth of 15.56% over OMIN 1, and 36.83% over OMIN 2.

Fig 7 reveals that the layout of industrial structure in Beijing influences the input of logistics system directly, and forms the different change in LMIN, IMIN, OMIN and RLIN factor. The IMIN occupies a leading role with a relative growth of 16.98% by 2022 in scenario (2), while OMIN is about 15.55% times less than the original value by 2022 in scenario (3). Government can recognize the inherent characteristics of logistics information management, and implement the feedback mechanism of coordinated development of Beijing’s economy and logistics system. Meanwhile, the industrial structure modes can be maintained, and the link between the logistics industry and other local industries should be strengthened.

### (2) Adjustment of system input

Since logistics system is influenced by the proportion of LMIN, RLIN, OMIN, IMIN, it’s crucial to gauge LSSC with two different strategies. Strategies (i): a certain index of factors such as the LMIN, RLIN, OMIN and IMIN will be modified by 1.5%, while the other indexes will be decreased by 0.5%. Strategies (ii): a certain index of the above parameters will be changed by 1.5%, while the other indexes will not be shown.

**Fig 8:**
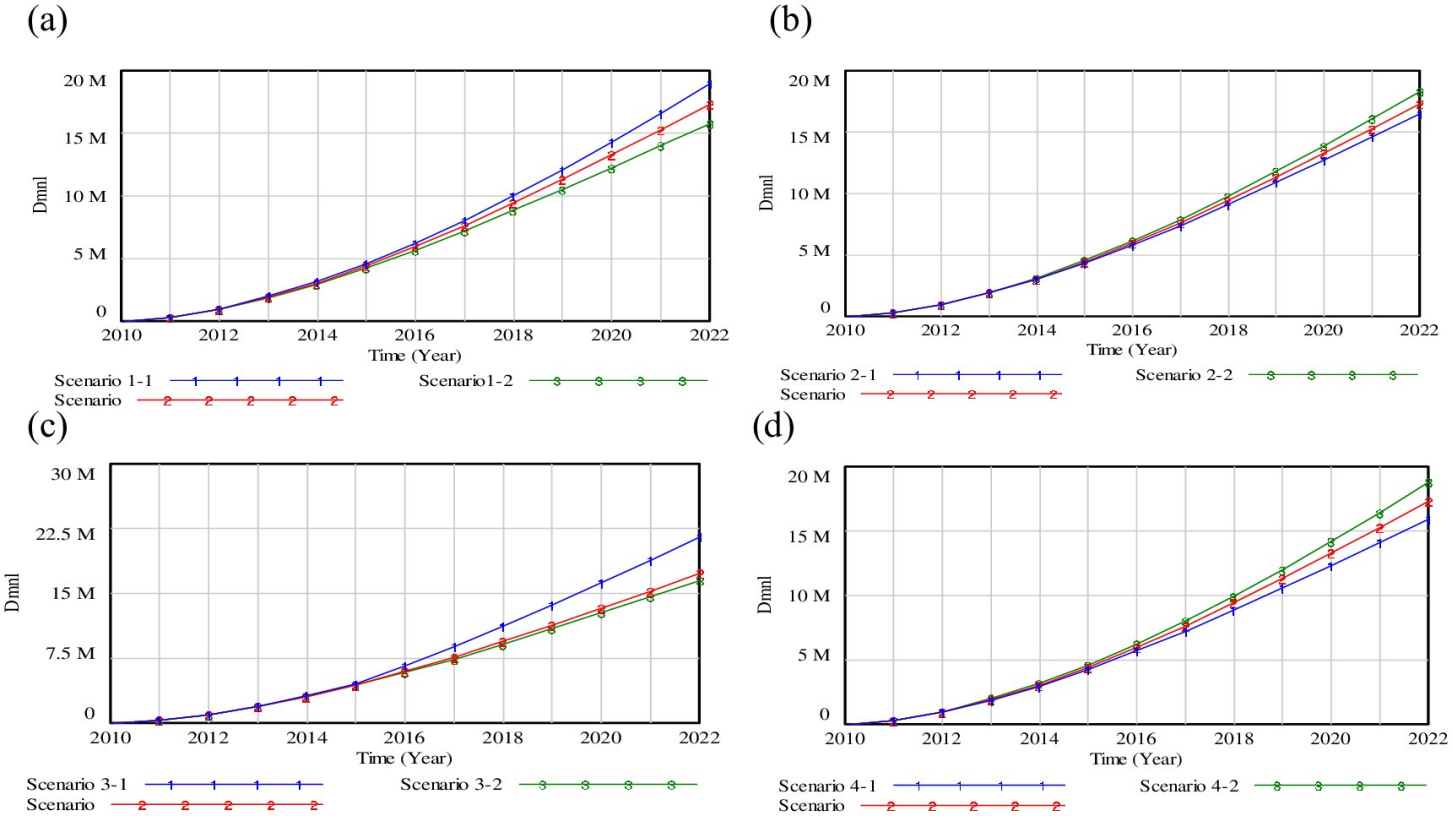
Results of strategies (i).

Fig 8(a) displays that the gap of LSSC was small from 2010 to 2015, and this gap gradually widened after 2016. LSSC in scenarios 1–1 would grow to 18932400 (Dmnl) in 2020, which can increase by 20.23% under scenarios 1–2. Fig 8(b) shows that the IMIN in 2022 will decrease by 9.92% under the scenario 2–1, with the comparison of scenarios 2–2. Result of RLIN can be seen in Fig 8(c). Scenario 3–1 may increase sharply to 21585400 (Dmnl) in 2022, while the value of RLIN in scenario 3–2 would decline by 4.62% compared to the base scenario. Fig 8(d) depicts that the OMIN under scenario 4–2 may reach to 18776400 (Dmnl) in 2022, if OMIN changes by 1.5%, while the others change by 0.5%.

We find that the results of scenario 3–1 are the best, whereas those of scenario 1–2 are the worst. Increasing the investment of IMIN or OMIN may not be sufficient to improve the value of LSSC. However, the investment of LMIN or RLIN would have a significant influence on the LSSC. Therefore, it is impossible to obtain the best logistics service by emphasizing only logistics management without involving reverse logistics management. The cooperation between recovery sectors is emphasized, since it strengthens the green logistics practice. We will pay particular attention to seeking the breakthrough point of shared technology and logistics system in Beijing.

**Fig 9:**
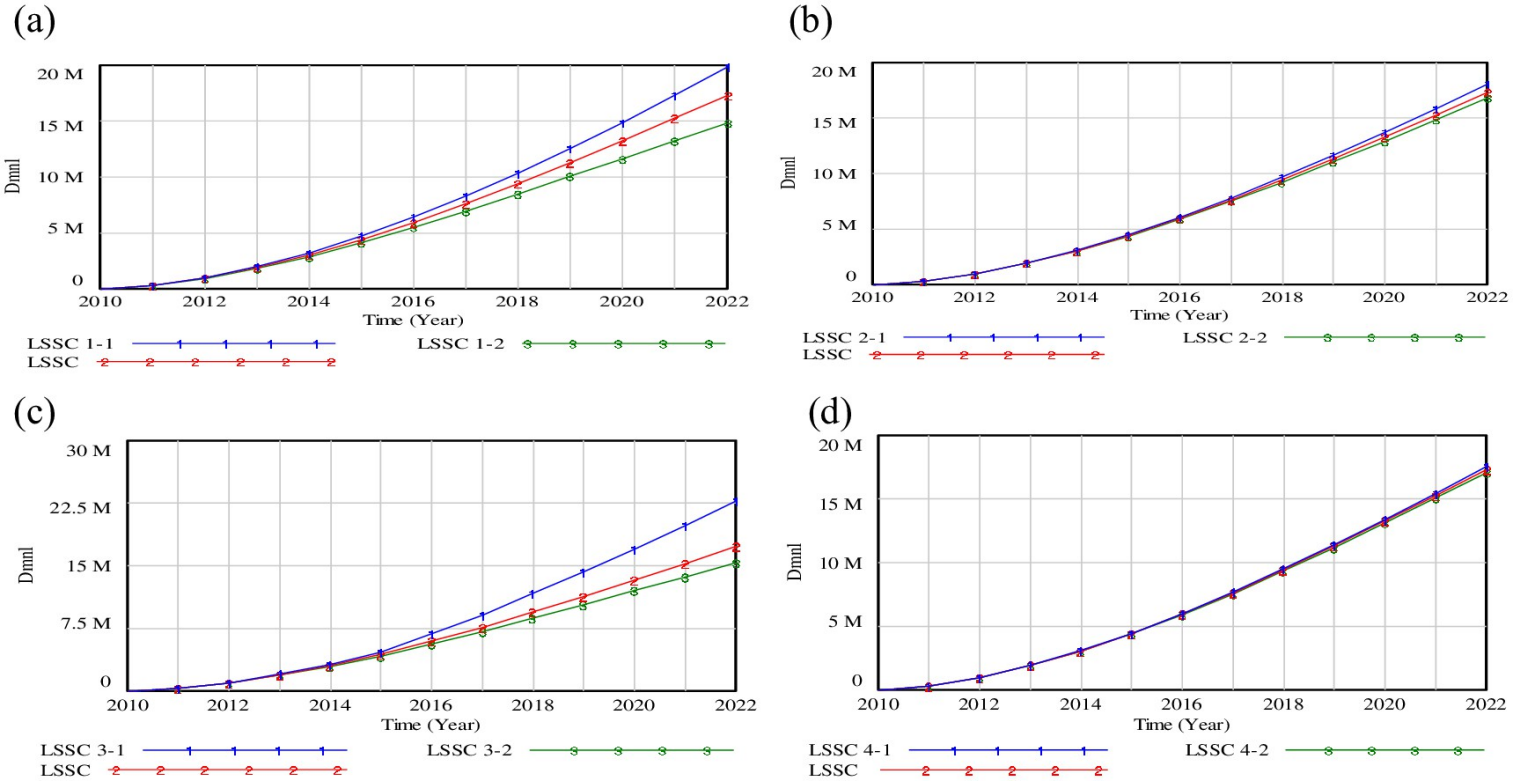
Results of strategies (ii).

To reveal the different influence of LMIN, IMIN, RLIN and OMIN on LSSC, a series of results for LSSC have been designed in Fig 9. Fig 9(a) depicts that if the input rate of LMIN is increased by 1.5%, while the others remain unchanged, LSSC will increase to 19852100 (Dmnl) in 2022. Otherwise, it will drop to 14826700 (Dmnl) by 2022, an increase of 11.88% over year. Fig 9(b) shows that IMIN helps the LSSC reach 18031200 (Dmnl) by 2022, which may increase by 7.36% compared with the strategy of decreasing the input rate. Fig 9(c) describes that the RLIN will rise to 22733600 (Dmnl) in 2022, under the scenario that only the growth rate of RLIN is changed. Results of the OMIN changes by 1.5% are depicted in Fig 9(d), which indicates a slight change of OMIN among the three scenarios.

On the whole, the result of LSSC 3–1 is the best, while that of the LSSC 1–2 is the worst. It indicates that increasing the input rate of RLIN alone or decreasing LMIN by the same multiple can have a significant impact on the LSSC. Some insightful suggestions are given for Beijing to build the logistics network, which is the “logistics base+logistics (distribution) center+terminal distribution”. Technologies are used to adjust organization patterns and the service patterns of green logistics, which are the desirable options for balancing the natural environment, energy demand and economic development.

Figs 8 and 9 show that the growth rate of LMIN, IMIN, RLIN, OMIN have different benefits on LSSC, and the influence of RLIN, LMIN, IMIN and OMIN on LSSC increases in turn. Although it is possibly related to coefficient setting of a few factors, the results may expose the internal mechanism to a large extent. Efforts should be made to enhance logistics service capability in Beijing such as cultivating the guidance for reverse logistics, promoting the deep integration of technology and system as well as developing new forms of green logistics. It is of utmost importance to increase the input of reverse logistics management, and establish the operation mechanism of reverse logistics.

### (3) Adjustment of transportation mode

**Fig 10:**
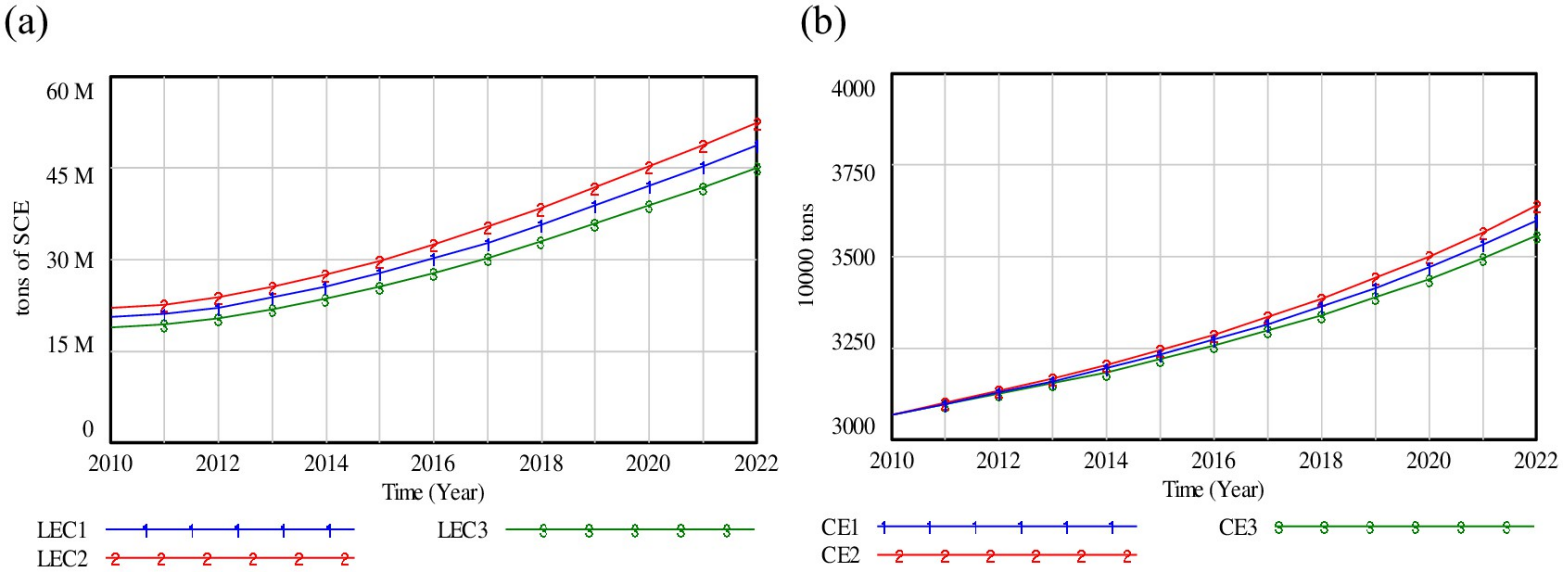
Simulation curves of the logistics energy consumption and carbon emissions. (a) LEC; (b) CE.

As the reduction of carbon emissions has been an urgent issue in developing a low-carbon economy, the impact of logistics energy consumption (LEC) on carbon emissions (CE) is substantially analyzed. The following policy scenarios can be created: scenario (1) displays the initial settings; scenario (2) shows that the percentage of highway transportation ascends by 6%, while the percentages of railway, civil aviation, and pipeline transportation decrease by 2%; scenario (3) and scenario (2) have opposite settings. Results are demonstrated in Fig 10.

Although the value of LEC has been increasing, its growth rate has slowed down from 2010 to 2014, as illustrated in Fig 10(a). The carve of LEC 2 will rise up to 18.287% in 2022 in comparison to LEC 3. Based on the above discussion, it is clear that the change of LEC will certainly affect carbon emissions. Fig 10(b) displays that the gap of CE carve remains unchanged in 2010 and 2011 years, and then this gap expands slowly. In 2022, results of CE2 may increase by 1.68% over the baseline scenario.

In comparison to the mode of civil aviation, pipeline and railway, highway transportation plays a key role in influencing the LEC and CE indexes. Statistical data presents that highway transportation within the dynamic system has a high sensitivity. To promote the long-term growth of logistics system, promulgating the policies of carbon emission control, and the carbon pricing and emissions trading are imperative [43]. It enables logistics managers to decide the optimal logistics recycling modes in accordance with carbon tax policy. Moreover, government should promote the publicity of low carbon awareness, and strengthen the implementation of green energy technologies. Companies can make more investment in low-carbon technology R&D, and increase the application of low-carbon technology, and pay more attention to emission-reduction technologies by building a carbon asset system.

## Conclusions and future research

According to the characteristics of the sustainability of logistics system such as dynamics and complexity, there is a lack of systematic evolution to reveal the interaction, feedback of factors within the system. Moreover, the obscured definition of system structure has made it difficult to evaluate the extensive development of the economy, environment, and logistics system of a given area. A set of boundaries involving the economy-operations subsystem, logistics carbon emission subsystem, and the logistics service subsystem are organized into a causal loop diagram, which is then converted to the stock and flow diagram of logistics system model. The following conclusions are drawn in detail below:

Firstly, the service capacity of logistics system (LSSC) and GDP factors have positive feedback loop in the process of a whole logistics system. Although there is a positive interaction between GDP and the freight traffic factors, surging logistics energy consumption is insufficient for the further progress of economy. Secondly, based on the validity and reality of logistics system model, results not only verify adjustment of the industrial structure has a direct impact on the system, but also reveal the different change in LMIN, IMIN, OMIN and RLIN factors. More specially, the IMIN indicator occupies a leading role can be explored. Furthermore, adjustment of system input shows that the LSSC is influenced by the investment in reverse logistics, logistics management, information and organizational management factor, and its degree of influence increases in turn. Finally, in comparison to the mode of civil aviation, pipeline and railway, highway transportation plays a key role in influencing the LEC and CE indexes. Adjusting transport mode depicts that model has a high sensitivity to the highway transportation.

Further work focuses on extending this model to the international cities to help them evaluate the impact of multiple policies on system. In order to properly meet the extensive needs of internal and external system elements, it is necessary to modify or add more parameters according to the various circumstances. Second, content analysis and expert consultations can assist in addressing the above limitation in the future. Third, the integrated model can be used to examine various scenarios, and address some issues about the long-term behavior of the intricate interactions between the environment, logistics system and economy.

## Supporting information

S1 Appendix Other factors and abbreviations of this model. (TIF)
