# Supplementary figures and images for: Towards the sustainable development of logistics system model: A system dynamics approach
Source: PLoS One. 2023 Jan 26;18(1):e0279687. doi: 10.1371/journal.pone.0279687 (PMC9879409; doi:10.1371/journal.pone.0279687)

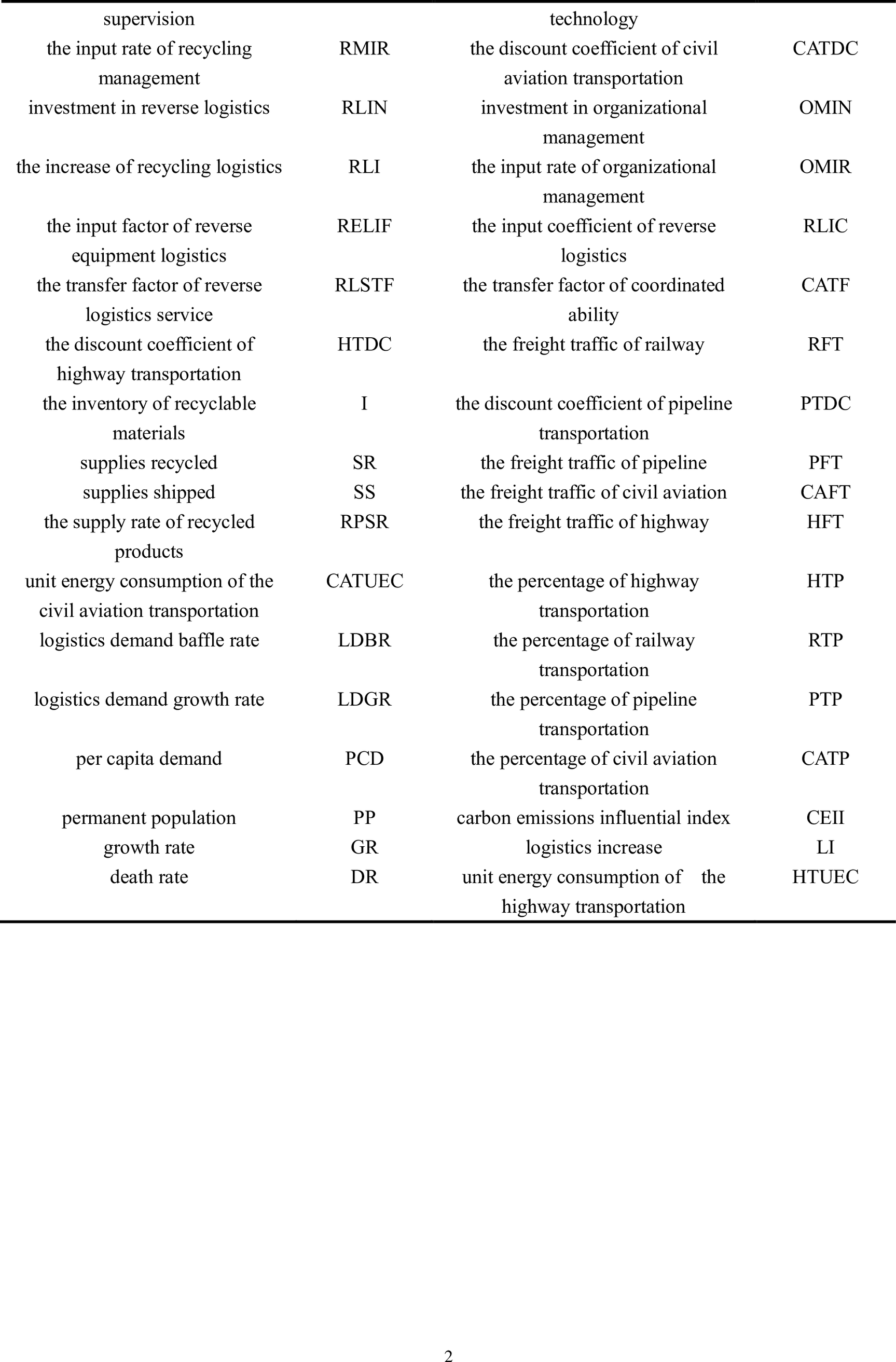

Supplement: S1 Appendix — (TIF) [file pone.0279687.s001.tif]
